# OAS1: A Protective Mechanism for Alzheimer’s Disease? An Exploration of Data and Possible Mechanisms

**DOI:** 10.3390/ijms26020524

**Published:** 2025-01-09

**Authors:** Richard J. Elsworthy, Alex Pearce, Farnoush Masoudzadeh, Klaudia Koska, Honey Lodhiya, Gargi Meher, Jodelle Adjej, Keeley J. Brookes

**Affiliations:** 1 School of Sport, Exercise and Rehabilitation Sciences, University of Birmingham, Birmingham B15 2TT, UK; r.j.elsworthy@bham.ac.uk; 2 Department of Biosciences, School of Science & Technology, Nottingham Trent University, Nottingham NG11 8NF, UK

**Keywords:** Alzheimer’s disease, OAS1, moderator, dsRNA, astrocytes

## Abstract

The immune system and neuroinflammation are now well established in the aetiology of neurodegeneration. Previous studies of transcriptomic and gene association studies have highlighted the potential of the 2′–5′ oligoadenylate synthetase 1 (OAS1) to play a role in Alzheimer’s disease. OAS1 is a viral response gene, interferon-induced, dsRNA activated enzyme, which binds RNase L to degrade dsRNA, and has been associated with COVID-19 response. This study explores whether a viral defence gene could play a vital role in neurodegeneration pathology. The genotyping of five SNPs across the *OAS1* locus was conducted in the Brains for Dementia Research (BDR) Cohort for association with AD. RNA-sequencing data were explored for differences in *OAS1* gene expression between phenotypes and genotypes. Finally, levels of dsRNA were measured in control cell lines, prior to and after exposure to amyloid oligomers and in cells harbouring a dementia-relevant mutation. No association of any of the *OAS1* SNPs investigated were associated with the AD phenotype in the BDR cohort. However, gene expression data supported the previous observation that the minor allele haplotype was associated with higher levels of the *OAS1* gene expression and the presence of an alternative transcript. Further to this, the presence of endogenous dsRNA was found to increase with exposure to amyloid oligomers and in the cell line with a dementia-relevant mutation. The data presented here suggest further exploration of the *OAS1* gene in relation to dementia is warranted. Investigations of whether carriers of the protective *OAS1* haplotype lower dsRNA presence and in turn lower inflammation and cell death are required to support the role of the gene as a moderator of neurodegeneration.

## 1. Introduction

Alzheimer’s disease (AD) is a complex disorder with combined genetic and environmental risk factors behind the aetiology of the neurodegeneration phenotype. The majority of cases present symptoms after the age of 65, showing progressive cognitive decline and memory loss [1]. With no cure available, most efforts are focused on prevention with the realisation that the accumulation of the disease hallmarks, β-amyloid (Aβ) plaques, and tau tangles begins years and perhaps decades before symptoms are recognised [2].

Whilst the harbouring of the APOE ε4 haplotype remains the greatest genetic risk factor for late onset AD, multiple genome-wide genetic association studies have highlighted the potential role of the innate immune system in the development of AD [3,4,5,6]. Microglia are the resident immune cells of the human brain that act to help to clear amyloid-β and regulate inflammatory processes. However, the over-activation of microglia is suspected to be key in the neuropathology of Alzheimer’s disease. The sustained pro-inflammatory environment is toxic to neurons leading to cell death and may drive the progression of symptoms associated with the disease [7]. Previous work utilising mouse models of AD overexpressing App and Psen1 gene mutations has identified a transcriptional network of almost 1600 genes that are differentially activated in microglia exposed to amyloid plaque deposition [8,9]. These include previously identified genes such as *Trem2*, *Abi3*, *Inpp5d*, *Plcg*2, and *Rin3*, as well as novel genes including *Oas1*, *Laptm5*, *Itgam*, and *Lilrba4*. Gene-based association tests utilising the IGAP dataset supported the potential role for these genes in AD pathology [9].

In a subsequent study, the group genotyped the rs1131454 single nucleotide polymorphism (SNP), indicated in the Genotype-Tissue Expression database (GTEx; https://gtexportal.org/) to regulate expression of the *OAS1* gene in the independent ARUK Alzheimer’s Disease DNA bank. This missense polymorphism located in the third exon of the gene indicated a significant association with the AD phenotype, indicating a protective effect of the minor G-allele (*p* = 0.0082, OR = 0.82; [10]). Further to this, the authors noted that OAS1 variants were also associated with COVID-19 response [11,12], and the SNPs were in strong linkage disequilibrium with the AD-associated SNP presented in their study [10]. In support of this, a further study investigating the allele frequency of the rs1131454 polymorphism with an incidence of AD across the globe also suggested that the G-allele was negatively correlated with AD [13]. Recent supporting evidence suggests that astrocytes carrying PSEN1 mutations demonstrate an upregulated inflammatory profile, which includes increases in *OAS1* expression [14].

The 2′–5′ oligoadenylate synthetase 1 (*OAS1*) gene, located on chromosome 12, encodes an interferon-induced dsRNA-activated enzyme, which binds RNase L to degrade dsRNA. Therefore, *OAS1* is known to be a part of the viral infection response; hence, its recent and robust association with the SARS-CoV-2 virus that causes COVID-19 with minor alleles across the *OAS1* haplotype is associated with a less severe response to COVID-19 [11,12].

Whereas the mis-sense polymorphism rs1131454 has not been observed to affect the activity of the gene [15], the splice acceptor polymorphism rs10774671, which is in high linkage disequilibrium with it, has received a great amount of attention due to the minor G-allele producing an alternative *OAS1* transcript. This G-transcript produces the p46 isoform, whereas the A-allele leads to the p48 and p52 isoforms [16]. The G-transcript has been shown to demonstrate higher expression and enzyme activity due to reduced vulnerability to nonsense-mediated decay [17]. The exploration of the GTEx database [18] supports this with the higher expression of *OAS1* associated with the minor allele of the rs10774671 as well as higher splicing rates in several tissues including the multiple brain regions. The p46 isoform of the OAS1 enzyme undergoes a post-translational modification of prenylation, which is thought to be responsible for augmenting its antiviral activity by targeting OAS1 to the endomembrane system; here, OAS1 clears dsRNA more efficiently, therefore, attenuating the inflammatory response [15,16,19].

Although *OAS1* expression is initiated by inflammation, the OAS1 protein is activated by the presence of dsRNA, attributed to viral infection. Therefore, despite the observation of increases in *OAS1* expression in dementia-relevant models, it may not be active. However endogenous dsRNA is also produced by cells, particularly when the cell is stressed, signalling that cellular processes are being mis-regulated [20]. Although not yet observed in AD, several other neurodegenerative disorders have demonstrated increases in endogenous dsRNA [21].

This study aims to strengthen the evidence that *OAS1* can act as a key modulator of the Aβ response and, therefore, a potential avenue to explore in relation to Alzheimer’s disease. The genotyping of *OAS1* SNPs was undertaken in the Brains for Dementia Research (BDR) project cohort, accompanied by the exploration of RNA-sequencing data quantifying gene expression and splice variants of *OAS1* in several samples from this cohort. Additionally, this study explored levels of dsRNA in human Neuronal-Progenitor-Cell-derived astrocytes exposed to Aβ oligomers.

## 2. Results

**Figure 1:**
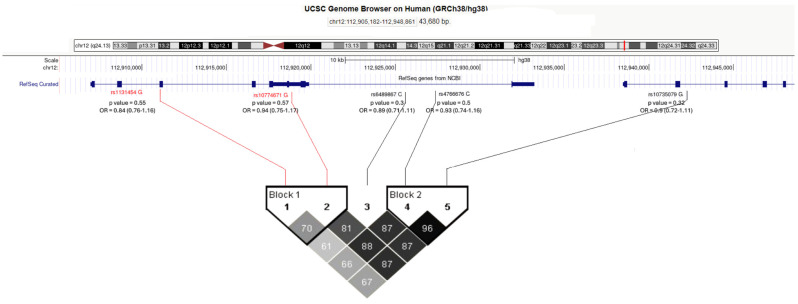
Schematic of investigated SNPs across *OAS1* locus from UCSC genome browser (hg38) overlaid with Haploview 4.1 software output calculated linkage disequilibrium (r2 values in boxes) between the SNPs. Association results are given next to rs SNP ID numbers, including minor allele, association significance value and Odds Ratio (95% Confidence Intervals). SNPs in red text indicate those indicated to have functional consequences according to GTEx database information. No association was observed with the Alzheimer’s disease phenotype in the Brains for Dementia Research Cohort; however, analysis using Haploview 4.1 software [22] did confirm high levels of linkage disequilibrium between the polymorphisms investigated.

From the BDR DNA bank, 445 samples were selected for analysis consisting of clinically diagnosed with AD-pathology-confirmed samples and 373 cognitively normal controls at death with no AD pathology. Known covariates for AD were explored and demonstrated significant differences between the case and control groups and, therefore, were included in the analysis (Table 1). No significant association was observed in this cohort, although strong linkage disequilibrium across the loci was observed (Figure 1), confirming the analysis of Magusali and colleagues (2021) [10] in a dementia-relevant cohort.

**Figure 2:**
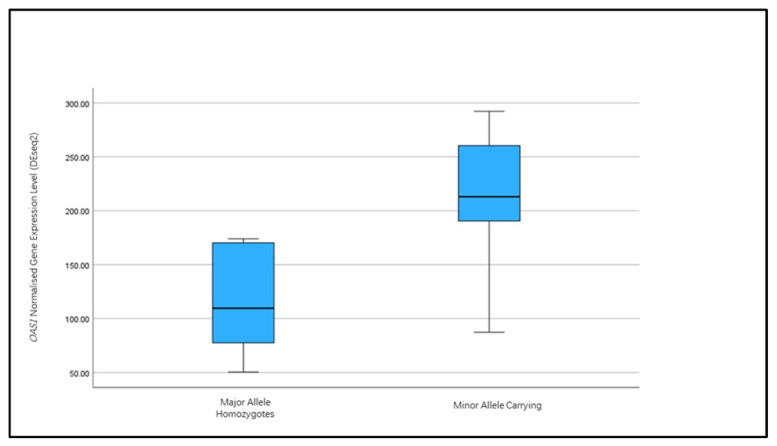
Box plots of normalised OAS1 gene expression from DESeq2 analysis. Samples homozygous for the A-allele for SNP rs10774671 (n = 10) were compared to those carrying the minor G-allele (n = 6). One-tailed *t*-test for mean expression between the groups indicated a significant difference (*p* = 0.005) in OAS1 expression, with those carrying the minor allele showing higher expression.

The exploration of gene expression analysis using DESeq2 described elsewhere [23] found no significant differential expression of *OAS1* between controls and Alzheimer’s disease in the post-mortem frontal cortex tissue. However, the exploration of normalised gene expression measures between samples homozygous for the major alleles of the SNP rs10774671 and those carrying the minor alleles (Figure 2) did indicate that the minor allele was associated with a higher expression of the *OAS1* gene (*p* = 0.005).

**Figure 3:**
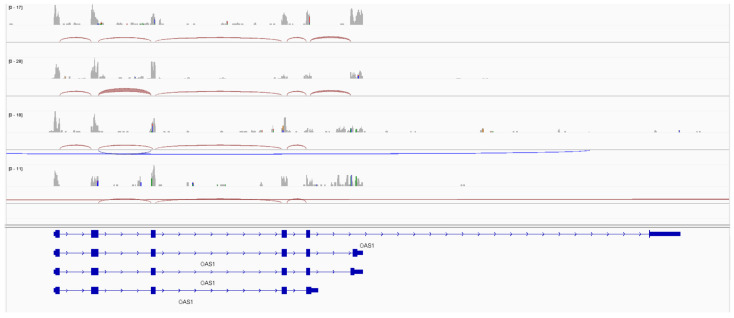
Aligned (hg38) RNA-sequencing data in Integrated Genome Viewer 2.12.2 [24] with OAS1 gene. Top two tracks show transcripts from two samples homozygous for the rs10774671 G-allele, and bottom two tracks show two samples homozygous for the rs10774671 A-allele. Splice junctions demonstrate alternative exon usage between the two genotypes.

The visualization of the RNA-sequencing data in the Integrated Genome Viewer 2.12.2 [24] demonstrated differential transcripts between samples that were homozygous for the minor G-allele to those who were homozygous for the A-allele (Figure 3), again confirming the previous literature that rs10774671 governed the alternative splicing of the *OAS1* gene in the post-mortem frontal cortex tissue.

**Figure 4:**
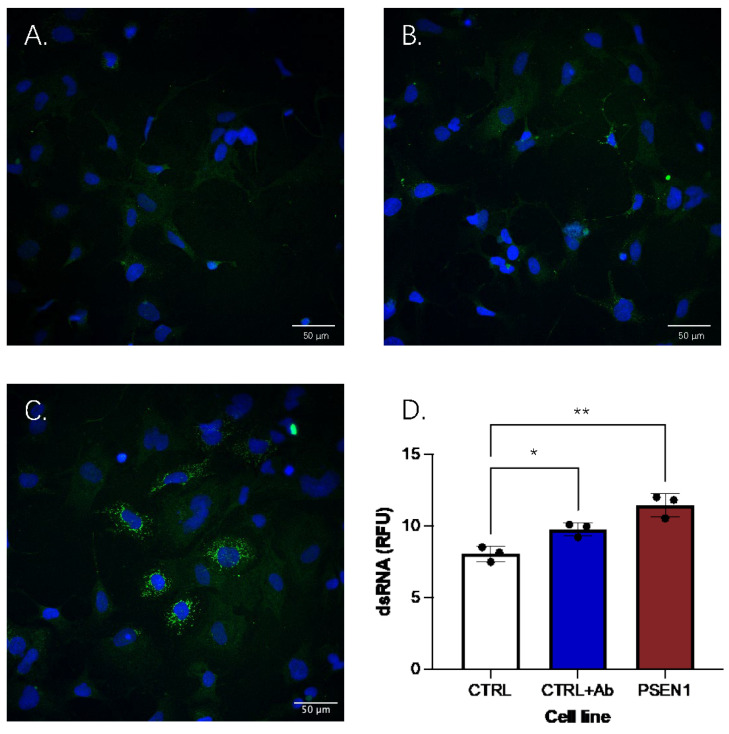
dsRNA in iPSC-derived astrocytes. Representative images shown for dsRNA measured using ICC in (**A**) healthy control astrocytes, (**B**) healthy control astrocytes treated with Aβ oligomers, and (**C**) astrocytes carrying a *PSEN1* mutation. (**D**) Average dsRNA levels including individual data points for replicates (averaged from 2 regions of interest) and error bars (SD) in hNPC-derived astrocytes. Measurements of dsRNA levels were higher in the control cells (CTRL) once exposed to Aβ oligomers (CTRL and Aβ; *p* = 0.028). Further to this, dsRNA levels were also elevated compared to controls in the *PSEN1* mutation carrying line (PSEN1; *p* = 0.001). * *p* < 0.05 and ** *p* < 0.001.

Despite the evidence that *OAS1* might be upregulated in AD due to the inflammatory profile, a crucial question is whether the enzyme is activated and how it could play a protective role. For the OAS1 enzyme to be activated, dsRNA must be present; therefore, it is key to explore whether increases in endogenous dsRNA are associated with amyloid deposition. The quantification of dsRNA in cells under resting conditions (Figure 4) indicated significant effects (F(2, 6) = 23.09, *p* = 0.002) compared to the exposure of the control cell line to Aβ oligomers (8.06 ± 0.52 RFU vs. 9.77 ± 0.47 RFU, *p* = 0.028) and in the *PSEN1* mutation line (8.06 ± 0.52 RFU vs. 11.46 ± 0.80 RFU, *p* = 0.001).

## 3. Discussion

This study aimed to provide further support and additional evidence for the role of the *OAS1* gene in Alzheimer’s disease. A gene association investigation with multiple markers across the *OAS1* locus in an independent AD cohort aimed to add supporting evidence for the previously observed genetic association with a protective effect from the minor alleles. Further to this, it sought to provide data for *OAS1* expression in AD diseased tissue to support previous observations of increased expression in response to Aβ, the hallmark of AD. Finally, the study aimed to add pertinent data to support a mechanistic pathway for the role of OAS1 enzyme in AD prevention by investigating levels of endogenous dsRNA levels in cells prior to and after Aβ oligomer exposure and in a cell line carrying a familial AD mutation.

This study did not replicate the genetic association observed in the previous studies [9,10,13] focusing on the eQTL SNP rs1131454. None of the five SNPs investigated displayed significant association with the AD phenotype in the BDR cohort; however, this study did support previous observations from the 1000 genomes study that a gene-wide haplotype did exist [10] and that this included a key SNP associated with COVID-19 response, with a clear mechanistic pathway to explain its biological effect. The BDR cohort is much smaller than other larger collaborative efforts conducting genome-wide association studies [3,5,6] and, therefore, lacks statistical power to observe alleles with small effect sizes. Given that the *OAS1* gene may be a moderator for AD pathology, the effect size would also be expected to be significantly smaller than main effect sizes. The strength of the BDR is the detailed neuropathology recorded on the post-mortem samples; therefore, as this sample increases in number, it may be feasible to re-investigate the *OAS1* association with the level of amyloid loading.

The indication of a role for the OAS1 enzyme in AD originally came to light through transcriptomic investigations in response to amyloid deposition in microglia from mouse models of AD [9] and subsequently supported by similar observations in iPSC-derived astrocytes from *PSEN1*-mutation-carrying cell lines [14]. Both of these studies observed a pro-inflammatory profile of gene expression, which included elevated *OAS1* expression, which is in line with the literature suggesting its expression is regulated by inflammatory signals [9,14]. This study sought to investigate the expression levels of the *OAS1* gene in a small (n = 16) post-mortem frontal cortex sample from participants of the BDR cohort. No observation of a significant expression difference for the *OAS1* gene was observed between the phenotypic groups. This was likely due to the small sample size available, and the exploration of an expanded dataset with information for amyloid loading (CERAD and Thal staging measures) and the *OAS1* genotype might prove more insightful.

Studies of *OAS1* in relation to COVID-19 response identified the association of rs10774671, an SNP governing alternative exon usage. The minor G-allele leads to an alternative transcript, which undergoes a prenylated post-translation modification of the enzyme, directing it to the endomembrane system [19]. The p46 isoform transcript also escapes nonsense-mediated decay and, therefore, appears to have higher gene expression levels [17]. The exploration of the human post-mortem brain RNA-sequencing data for *OAS1* confirms that carriers of the minor allele do show higher levels of *OAS1* gene levels compared to the homozygous A-allele samples and that the comparison of homozygote samples shows differential exon usage aligned with the different isoforms of the OAS1 enzyme.

A further area of exploration might be to explore the functionality of the haplotype between the splicing SNP rs10774671 and the eQTL SNP rs1131454. Is the perceived increase in the expression of *OAS1* attributed to rs1131454 merely due to the level of linkage disequilibrium with the alternative transcript that escapes nonsense-mediated decay? Or is this effect independent? The observed breakdown of linkage disequilibrium compared with the other SNPs in the haplotype for rs1131454 might indicate the latter is worth investigation.

Importantly, this study adds data to fill the mechanistic gap for the potential role for OAS1 in AD pathology, providing the first evidence that endogenous dsRNA is produced in response to Aβ oligomers and indicating that OAS1 might dimerize and facilitate RNA clearance, reducing inflammation. However, further research to directly measure alterations in OAS1-mediated pathways is needed. This provides support for a biological mechanism behind the protective association of the *OAS1* minor alleles with AD pathology, where carriers of the minor alleles can clear dsRNA more effectively and reduce neuroinflammation in the brain, perhaps delaying neuronal death. A direct comparison of cellular phenotypes in relation to dsRNA and neuronal death between *OAS1* genotypes is now warranted to test this hypothesis. Additionally, whether the dsRNA response to Aβ oligomers is the same across multiple healthy control lines and in cells carrying different *PSEN1* mutations needs further investigation.

A further question to ask is where the dsRNA is coming from, and why might the p46 isoform of OAS1 directed to the endomembrane system has a more robust effect. The SARS-CoV-2 replication occurs within organelles of the endomembrane system; therefore, the prenylated p46 isoform of OAS1 is directed to the membranes and can mediate the degradation of the viral RNA at the point of replication [12]. Whilst most endogenous cellular dsRNA is primarily released from the mitochondria, which are not seen as a part of the endomembrane system, other sources of endogenous dsRNA may also come direct from the nucleus. Aberrant dsRNA expression may come from this organelle of the endomembrane system in the form of transposable elements, long interspersed nuclear elements (LINEs) and short interspersed nuclear elements (SINEs), which have escaped epigenetic repression [20]. Interestingly, the expression of the endogenous retroviral class of transposable elements has been observed to be increased in AD in relation to Tau pathology [25].

This study suggests that the role of the OAS1 enzyme in relation to AD warrants further exploration. It is likely that OAS1 may not be a causal risk factor in neurodegenerative pathology but a moderator, influencing the immune response to Aβ, supporting hypotheses that AD may be due to an inadequate immune response to the accumulation of amyloid in the brain [26].

We hypothesise that excess amyloid stresses the cell, leading to an increase in cellular dsRNA. This activates OAS1, leading to the clearance of the dsRNA. The presence of the p46 isoform leads to greater levels of clearance, limiting the pro-inflammatory response, which has been attributed to the neuronal cell death observed in AD. Therefore, we propose that carriers of the minor alleles of *OAS1* will demonstrate a quicker response to amyloid deposition, the clearance of dsRNA, the lowering of inflammation, and reduced cell death.

## 4. Materials and Methods

Samples: The Brains for Dementia Research (BDR) project is an established semi-longitudinal programme that provides a wealth of information for researchers investigating dementia, which includes post-mortem brain tissue donations [27]. Alongside the cognitive, lifestyle, and neuropathological detail obtained during life and upon death, DNA has been extracted from samples of post-mortem brain tissue to create a DNA bank for research purposes, and whole-genome data are also freely available for scientific exploration [28].

The DNA bank currently stands at 1127 samples from deceased participants for whom a diagnosis has been made based on clinical and neuropathological features for genetic analyses. This cohort contains a mix of different dementias including AD, Vascular Dementia, Dementia with Lewy Bodies, and Frontal Temporal Lobe Dementia alongside mixed pathologies, those with Mild Cognitive Impairment, and cognitively normal controls. For this study, only participants with neuropathological-confirmed (clinical diagnosis of dementia and AD pathology present) AD (n = 445) and controls without cognitive deficits or neurodegenerative comorbidities (n = 373) were analysed. Details on the demographics for key AD covariates can be found in Table 1, with all covariates suggesting a significant difference between the groups on ratio of females, age at death, and presence of the APOE ε4 isoform.

Genotyping: This study aimed to replicate the original findings of association for the *OAS1* gene with AD, using the Brains for Dementia Research (BDR) cohort. Genotyping of five SNPs across the locus was conducted; these included the SNPs identified from AD studies, rs1131454 [10,13] and rs4766676 [9]; the SNPs identified to be associated with COVID-19 response, rs6489867 and rs10735079 [10]; and the SNP associated with COVID-19 response and governing alternative splicing of the OAS1 gene rs10774671 [12]. In-house genotyping of the polymorphisms was conducted using TaqMan assays following standard protocols (Applied Biosystems, Waltham, MA, USA). Quantitative PCR reactions were run on the Aria Mx real-time PCR machine (Agilent Technologies, Santa Clara, CA, USA). Association analysis was carried out in PLINKv1.9 [29]. Individual SNP association analysis was carried out using a logistic regression test correcting for the following covariates: biological sex, age at death, and APOE ε4 allele count.

RNA-sequencing data: Several BDR samples have been RNA-sequenced and explored for differential expression and eQTL analysis presented elsewhere [23]. Briefly, sixteen samples were selected from the BDR cohort (Oxford Brain Bank’s generic REC approval 15/SC/0639) for RNA-sequencing. All samples were neuropathologically confirmed AD cases (n = 8) or cognitively normal controls (n = 8) with no other neuropathology. Samples were matched on biological sex, age at death (*p* = 0.69), and PMI (*p* = 0.67), and all samples were homozygous for the APOE isoform ε3. RNA was extracted from bulk frontal-cortex tissue using previously established protocols [30]. An amount of 20 ng of total RNA per sample was provided to the UCL Genomics Facility (London, UK) for Kapa mRNA HyperPrep library preparation and sequenced on the Illumina NextSeq 2000, generating ~30 M paired end reads per sample for analysis. These data were used to explore both *OAS1* gene expression and alternative transcript present in relation to genotype.

### 4.1. Cell Culture and Astrocyte Differentiation of Human Neural Stem Cells

Healthy control (ax0018) and familial AD (fAD) A246E *PSEN1*-mutated (ax0114) human Neuronal Progenitor Cells (hNPCs) were purchased from Axol Bioscience (Table 2, Cambridge, UK). To generate astrocytes, following previously described methods, hNPCs were differentiated into astrocytes [31].

Briefly, hNPCs were seeded at a density of 7 × 10^4^ cells/cm^2^ in neural plating medium (Axol Bioscience, Cambridge, UK) on Matrigel matrix (356237, Corning, Corning, NY, USA)-coated wells. After 24 h, cells were washed with D-PBS before medium was exchanged for astrocyte differentiation medium (ADM) (STEMdiff Astrocyte™ differentiation kit #100-0013, StemCell Technologies, Cambridge, UK), and a full media exchange was completed every day for 7 days. Cells were passaged using 1 mL/well Accutase™ (A6964, Merck, Feltham, UK), and dissociation was stopped with 4 mL/well ADM. Cells were maintained in a 37 °C, 5% CO_2_/95% air atmosphere with a total medium exchange every other day, through two subsequent passages, before switching to astrocyte maturation medium (STEMdiff Astrocyte™ maturation kit #100-0016, StemCell Technologies, Cambridge, UK). Media were changed every other day for 7 days before cells were passaged as before and cultured in astrocyte maintenance media (ScienCell astrocyte media, Cat #1801, Carlsbad, CA, USA) until day 45. Astrocytes were switched to BrainPhys™ Neuronal Medium supplemented with SM1 (StemCell Technologies, Cambridge, UK) for 5 days prior to experiments.

### 4.2. Immunocytochemistry

To confirm the successful differentiation of hNPCs into astrocytes, immunocytochemical analysis of astrocyte markers were imaged. This has been described in previous work [31]. Briefly, cells were fixed in 4% (*v*/*v*) paraformaldehyde (PFA) in Dulbeco’s Phosphate-Buffered Saline. The cells were then incubated for 10 min in PBS with 0.2% (*v*/*v*) Triton X-100, followed by blocking for 1 h in PBS containing 0.2% (*v*/*v*) Triton X-100 and 3% (*w*/*v*) bovine serum albumin (A9418 Sigma-Aldrich, Dorset, UK). Primary antibodies for Aldehyde Dehydrogenase 1 Family Member L1 (ALDH1L1) (702573, Invitrogen), glial fibrillary acidic protein (GFAP) (14-9892-82, Invitrogen, Waltham, MA, USA), and S100β (PA5-78161, Invitrogen, Waltham, MA, USA) were diluted in blocking buffer and added for 1 h. Cells were then washed with blocking buffer and appropriate secondary antibodies. Alexa Fluor^®^ 488 AffiniPure Goat Anti-Rabbit IgG (1:2000, 111-545-144, Jackson Laboratories, London, UK) and Alexa Fluor^®^ 633 Goat Anti-Mouse IgG (1:2000, A-21052, ThermoFisher Scientific, Cambridge, UK) were then added for 1 h. Cells were incubated for 15 min DAPI (P3935, ThermoFisher Scientific, Cambridge, UK) before 3× wash with D-PBS and then mounted with ProlongTM Gold Antifade Mountant (P36930, Invitrogen, Paisley, Renfrewshire, UK) to glass slides. After incubation at room temperature overnight, slides were imaged using a Zeiss LSM 780 confocal microscope (Oberkochen, Germany).

### 4.3. Preparation and Treatments of Synthetic Aβ1-42 Oligomers

Human hexafluoroisopropanol (HFIP) Aβ1-42 (AG968, Sigma-Aldrich) was prepared in oligomeric form. Briefly, human HFIP Aβ1-42 was resuspended in DMSO to 5 mM. Monomers were diluted in F-12 culture media, without phenol red, to a concentration of 100 μM and incubated for 24 h at 4 °C. Confirmation of oligomerisation and cellular uptake have been described in previous work [31]. To determine the possible effects of human Aβ1-42 oligomers on inducing dsRNA in both astrocytes and microglia, cells were treated with oligomeric Aβ1-42 (1 μM). Cells were incubated for 48 h at 37 °C in a humidified atmosphere of 5% CO_2_.

### 4.4. Quantification of dsRNA

To quantify dsRNA in cells, ICC with confocal microscopy was performed as described above. The anti-dsRNA monoclonal antibody J2 (RNT-SCI-10010200, Jena Bioscience, 1:200) was used to detect dsRNA following treatment with oligomeric Aβ1-42 (1 μM for 48 h). Alexa Fluor^®^ 568 Goat Anti-Mouse IgG (1:2000, A-11004, ThermoFisher Scientific) was then added for 1 h before cells were incubated for 15 min DAPI (P3935, ThermoFisher Scientific, Cambridge, UK), washed in DPBS, and then mounted with ProlongTM Gold Antifade Mountant (P36930, Invitrogen, Waltham, MA, USA) to glass slides. After incubation at room temperature overnight, slides were imaged using a Zeiss LSM 780 confocal microscope. For quantification, images from 3 replicates were captured and averaged across 2 regions of interest per replicate and analysed using Image J v1.53. Background ROIs were subtracted from mean pixel intensity across each panel.

## 5. Conclusions

The *OAS1* gene has been genetically associated with lower risk for AD and has been observed to show increased expression in response to Aβ in previous studies. However, the canonical function of this gene is to limit viral infection by facilitating the removal of dsRNA, making its link with AD uncertain. This exploratory study combines different strands of inquiry to develop a working hypothesis of how the OAS1 enzyme might exert a protective effect on AD pathology. Despite the sample size of the genetic cohort being too small to support previous observations of a genetic association, the study demonstrates high linkage disequilibrium with a splice site variant that is known to have favourable outcomes on viral infections. This study demonstrates that carriers of the protective variants have elevated levels of *OAS1* gene expression in human post-mortem brain, which is subject to neuroinflammation in AD. This study also shows that the protective variant does lead to alternative splicing aligned with the production of the P46 OAS1 isoform, which is shown to be more effective in the removal of dsRNA and attenuation inflammation. In the absence of viral infection, cells often produce dsRNA as a stress response, and the accumulation of Aβ may be considered a stressor, causing inflammation, which increases the expression of *OAS1*. This study demonstrates that the presence of Aβ also causes an increase in endogenous dsRNA, which activates the OAS1 enzyme; therefore, the benefits of carrying the more effective *OAS1* genetic variants against viral infection may also afford a protective moderator response to Aβ and consequently AD.

Future studies should focus on whether carriers of the protective variant of *OAS1* show a better/quicker clearance of dsRNA/inflammation and if this results in less neuronal cell death in the presence of Aβ compared to the major variant; and whether it is the higher levels of *OAS1* afforded by the alternative transcript escaping RNA decay or the prenylation modification of the resulting P46 isoform of OAS1 that is the critical factor.

## Figures and Tables

**Table 1:** Demographics of the Alzheimer’s disease (n = 445) and control (n = 373) samples explored for association in this study. Known covariates with the phenotype, biological sex, average age at death (standard deviation, sd), and presence of the APOE ε4 isoform were all significantly different between the AD and control groups.

	Controls (n = 373)	AD (n = 445)	*p* Value
Percentage female	56.8%	48.9%	0.025
Mean age at death	86.3 years (sd = 8.6)	83.4 years (sd = 8.6)	<0.00001
APOE-ε4-positive	25.7%	68.9%	<0.00001

**Table 2:** Information of the human Neuronal Progenitor Cell lines used to explore dsRNA levels. Adapted from [18]. Not Applicable (n/a).

	Ax0018 (Control)	Ax0113 (fAD)
Diagnosis	None	Familial AD
Sample type	Dermal fibroblast	Dermal fibroblast
Donor sex	Male	Female
Age at sampling	74 years	31 years
Age at onset	n/a	45 years
Karyotype	Normal	Normal
Reprogramming	Episomal vector	Episomal vector
Induction method	Monolayer—Axolbio NIM	Monolayer—Axolbio NIM
Mutation	None	PSEN1 (A246E)
APOE status	ε2/ε2	ε2/ε3

## Data Availability

Genetic data and raw RNA-sequencing reads are available via the Dementias Platform UK Data Portal (https://portal.dementiasplatform.uk/).
